# Understanding CAM Use in Lebanon: Findings from a National Survey

**DOI:** 10.1155/2018/4169159

**Published:** 2018-07-25

**Authors:** Samer Kharroubi, Rana F. Chehab, Chirine El-Baba, Mohamad Alameddine, Farah Naja

**Affiliations:** ^1^Department of Nutrition and Food Sciences, American University of Beirut, Riad El-Solh, Beirut 1107 2020, Lebanon; ^2^Department of Nutrition Science, Purdue University, West Lafayette, IN, USA; ^3^Mohammed Bin Rashid University of Medicine and Health Sciences, Building 14, Dubai Healthcare City, 505055 Dubai, UAE; ^4^Department of Health Management and Policy, Faculty of Health Sciences, American University of Beirut, Riad El-Solh, Beirut 1107 2020, Lebanon

## Abstract

The main objective of this study was to identify predictors of Complementary and Alternative Medicine (CAM) use in Lebanon. Data for this study were drawn from a national survey conducted among Lebanese adults (n=1500). A modified version of the Social Behavioral Model (SBM) was used to understand CAM use in the study population. In this version, predisposing factors included sociodemographic characteristics (age, gender, education, and employment) and* Push* and* Pull* factors. Additionally, enabling resources included income, and medical need encompassed presence of chronic disease and perceived health status. Simple and multiple logistic regressions were used to examine the predictors of CAM use in the study population. Results of the multiple logistic regression showed that younger and older adults were less likely to use CAM as compared to middle-aged respondents. The* Push* factor “dissatisfaction with conventional medicine” was associated with higher odds of CAM use. For three of the six* Pull* factors, compared to participants who strongly disagreed, those who had a tendency of taking care of one's health were more likely to use CAM. Income and presence of chronic disease were also associated with higher odds of CAM use. The findings of this study affirmed the utility of the SBM in explaining the use of CAM and proposed a new version of this model, whereby the* Push* and* Pull* factors are integrated within the predisposing factors of this model.

## 1. Background

In recent years, there has been a global renaissance of interest in natural and herbal remedies. This is partly due to the realization that conventional medicine is not capable of providing a cure or solution for human diseases and that the presence of side effects is almost unavoidable [1–3]. Complementary and alternative medicine (CAM) is defined by the National Institutes of Health as “a group of diverse medical and health care systems, practices, and products that are not generally considered part of conventional medicine” [4]. Worldwide, the prevalence of CAM use varies between 10% and 76% [5] and is highest among patients with chronic diseases [6]. According to CAMbrella, a European research network for CAM, herbal and biological-based medicine was the most common CAM reported in Europe [7]. Such a preponderance of CAM use has not been accompanied by a parallel growth in the scientific evidence to ascertain its safety and efficacy [8].

The increasing prevalence rates of CAM use coupled with the potential side effects of certain CAM modalities and their negative interactions with several conventional treatments sparked interest in examining and understanding the drivers of CAM use in various populations, from academic as well as applied perspectives [9]. A number of conceptual models originating from various fields including medical sociology, psychology, and marketing were studied in this context [10].

The Social Behavioral Model (SBM), first developed in 1968 by Andersen, is among the most commonly used models to predict healthcare utilization behavior, including CAM use [11]. The core of this model is centered on three pillars as determinants of healthcare utilization: (1) predisposing factors, (2) enabling resources, and (3) medical need. The predisposing factors include, in addition to sociodemographic characteristics, health beliefs and social values. The enabling resources relate mainly to income and financial ability to access healthcare. As for medical need, it includes both self-perceived and evaluated health status [12]. While medical need constitutes a direct motivator for CAM use, predisposing factors such as health beliefs play a relatively indirect role in this behavior [13].

Health beliefs as potential determinants for CAM use were grouped into either the reasons that underscore the negative aspect of conventional medicine or* Push* factors or the reasons that related to the desire of a more proactive role in one's health and holistic health beliefs or* Pull* factors [12, 14–16]. The* Pull* factors, in contrast, were more focused on the beliefs that CAM use is likely to confer benefits in overall health, energy, immunity, and quality-of life and stimulate feelings of hope, enhance participation in or mastery of one's own health, and/or offer an alternative to conventional medicine that is holistic, natural, and congruent with one's life philosophy [17]. The majority of research studies that investigated the* Push* and* Pull* factors in relation to CAM use originated in Europe, Canada, and the United States and yielded inconsistent findings [12, 16–19].

In Lebanon, a small country of the Middle East and North Africa (MENA) region, a national survey revealed that one in three Lebanese adults uses CAM, with herbs being the most commonly used type [20]. Such a prevalent use of CAM is not coupled with national regulatory frameworks that optimize public safety and support the proper integration of CAM into the healthcare system [21]. Therefore, the main objective of this study was to identify the factors that predict CAM use based on national data from Lebanon by applying the three constructs of the SBM: predisposing characteristics (sociodemographics), enabling resources (income), and medical need (health status). Health beliefs including the* Push* and* Pull* factors were also studied as predisposing factors within the SBM. A secondary objective was to investigate the effect of various sociodemographic characteristics on the* Push *and* Pull* factors.

## 2. Methods

### 2.1. Study Population

Data for the present study were drawn from a cross-sectional survey conducted on a nationally representative sample of Lebanese adults. Data collection took place between August 2010 and January 2011. A stratified cluster random selection design was used to attain a nationally representative sample. Details about the sampling frame and selection of participants are described elsewhere [22]. Participation was voluntary, and informed consent was obtained from all respondents prior to participation. The average administration time of the interviews was 20 minutes. The study protocol was approved by the Institutional Review Board at the American University of Beirut (protocol number FHS.MA.03).

### 2.2. Theoretical Constructs

**Figure 1:**
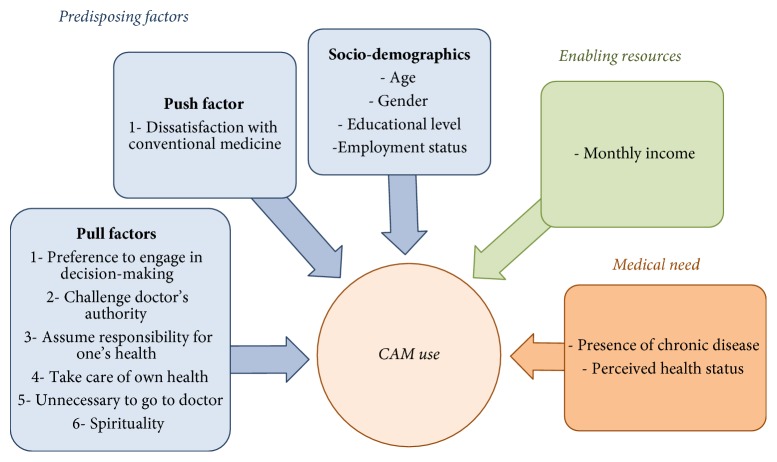
A modified version of the Social Behavioral Model for CAM use among the Lebanese population.

For the purpose of this study, CAM referred to “biologically based practices” and included dietary supplements, herbal products, and the other so-called natural yet scientifically unproven therapies [4]. As previously highlighted, the SBM includes three major constructs: the predisposing, enabling, and medical need factors. In this study, predisposing factors included age, gender, education, and employment, in addition to health beliefs (*Push* and* Pull* factors). Measures of enabling resources included income, while those of medical need included presence of chronic disease and perceived health status. The modified SBM used in this study is depicted in Figure 1.

### 2.3. Survey Instrument

Respondents completed a multicomponent questionnaire through face-to-face interviews. The interviewers were extensively trained on the administration of the questionnaire to have a nonjudgmental attitude and not to provide leading questions. The questionnaire used in data collection was developed by the research team and thoroughly reviewed by an expert panel consisting of a medical doctor, an epidemiologist, a health management and policy expert, and an economist. The questionnaire explored the sociodemographic and health-related characteristics of respondents, their use of CAM during the previous 12 months, and their beliefs related to CAM use, that is,* Push* and* Pull* factors. Regarding the* Push* factors, patients were asked about their dissatisfaction with conventional medicine during the past 12 months (yes or no). The* Pull* factors were assessed through a series of six statements that the respondents were asked to rate on a 5-point Likert scale ranging from 1 (strongly disagree) to 5 (strongly agree). The statements were as follows: “I prefer that doctors give me choices or options and let me decide for myself what to do” (*Pull* factor 1); “Patients should challenge the authority of the doctor” (*Pull* factor 2); “I prefer to assume some of the responsibility” (*Pull* factor 3); “Except for serious illness, it is better to take care of one's own health than go to the doctor” (*Pull* factor 4); “It is not obligatory to go to the doctor to treat oneself” (*Pull* factor 5); and “Spirituality and faith play important roles in life” (*Pull* factor 6).

The questionnaire was pilot-tested on 35 randomly selected individuals who were asked to provide feedback on the clarity and flow of the questionnaire. The received feedback was incorporated in the final version of the questionnaire.

### 2.4. Statistical Analysis

Data entry and analysis were conducted using the Statistical Package for the Social Sciences (SPSS) software version 24 for Windows [23]. Frequencies and percentages were used to describe characteristics of CAM use. Differences between CAM users and nonusers with regard to sociodemographics, health variables, and* Push* and* Pull* factors were all assessed using Pearson's Chi-square tests. The associations of each SBM construct (SES, health status, and* Push* and* Pull *factors) with CAM use were assessed using simple logistic regression, with CAM use being the dependent variable. In order to evaluate the correlations of CAM use, a multiple logistic regression model was utilized. In this model, variables were included if they were significantly associated with the dependent variable in the univariate analysis. Similarly, the associations of SES and health status with the* Push* factor were also assessed using simple and multiple logistic models, with feeling dissatisfied from conventional medicine being a dependent variable. The associations of various sociodemographic and health characteristics with each of the* Pull* factors were examined using ordinal regression analysis. Each* Pull* factor measured on the 5-item scale was used as the ordinal dependent variable. In order to adjust for possible confounders, a multiple ordinal regression model was built, in which all sociodemographic and health characteristics were used as independent variables. Odds ratios and their respective 95% confidence intervals were computed. While a p-value of 0.05 was used to detect significance in simple regression analyses, a more conservative value (0.01) was considered for the multiple regressions used in this study.

## 3. Results

### 3.1. Data on the SBM Factors in the Study Population

Table 1 displays data on the SBM constructs representing sociodemographic and health-related characteristics of the overall study population including CAM users and nonusers. The sample population comprised respondents from all three age groups: ≤31 years (36.2%), 32-51 years (39.2%), and ≥52 (24.6%), with 816 (54.4%) males and 684 (45.6%) females.

### 3.2. Push and Pull Factors and CAM Use

Table 2 shows the associations between the* Push* and* Pull* factors and CAM use among the study respondents. CAM users were significantly more dissatisfied with conventional medicine compared to nonusers. The percentage of respondents by level of agreement/disagreement is also shown in Table 2. Overall, the majority of the respondents strongly disagreed with all* Pull* factors except the sixth factor on “spirituality and faith.” However, there were significant differences between CAM users and nonusers in their levels of agreement on the first five* Pull* factors (*Pull* factors 1-5). CAM users were less likely to “strongly disagree” with these five* Pull* factors, in comparison to nonusers.

Table 3 represents the simple and multiple logistic regression analysis of the SBM constructs and the* Push *and* Pull *factors of CAM use in the study population. Using results from the simple logistic regression, variables significantly associated with CAM use in the study population included age, education, monthly income, presence of chronic disease, the* Push* factors, and the first five* Pull* factors. In the multiple logistic regression, variables were put in the model in order of strength of their association with CAM use as per the simple logistic analysis. The effect of each variable on the model was assessed and the variable was kept if it significantly contributed to a better fit of the model. The final multiple logistic model included the following variables: age, monthly income, presence of chronic disease, the* Push* factors, and three out of the six* Pull* factors (1, 4, and 5). The results of the multiple logistic analysis showed that CAM use was significantly associated with age (p<0.01), monthly income (p<0.01), the* Push* factors, and* Pull* factors 1, 4, and 5. Finally, presence of chronic diseases was also associated with CAM use, with borderline significance (p=0.018).

### 3.3. The Push Factors and Sociodemographic Characteristics

Simple and multiple logistic regression models were created to explore the association of sociodemographics with the* Push* factors (Table 4). In the simple model, age (≤31 years old), females, high school and university education, middle- and high-income groups, presence of chronic disease, and poor-to-good perceived health status were all significantly associated with the use of CAM. The multiple logistic model included the following variables: age, monthly income, presence of chronic disease, and perceived health status. The multiple analysis indicated that the odds of feeling dissatisfied with conventional medicine were significantly associated with age and monthly income. On the other hand, the odds of feeling dissatisfied with conventional medicine were lower among respondents reporting having chronic disease and those who perceived their health status as good, fair, and poor.

### 3.4. Pull Factors and Sociodemographic Characteristics

Ordinal logistic regression models were used to examine the associations of various sociodemographic and disease characteristics with each of the* Pull* factors in the study population (Appendix A). In the regression model, each of the* Pull* factors was used as the ordinal dependent variable, while sociodemographic and disease characteristics were used as independent variables. To determine the factors associated with* Pull* factor 1, variables that met significance in the simple analysis were entered in the final multiple model. Similar ordinal regression models were applied for the other* Pull* factors, that is,* Pull* factors 2-6. Appendix A displays the parameter estimates table, which includes the coefficients, the 95% confidence interval of the coefficients, and their associated* p*-values for each of the* Pull* factors. Note that a positive (negative) coefficient means that a higher (lower) value/score in the ordinal dependent variable is more likely. A summary of the significant associations among sociodemographic characteristics and the various* Pull* factors, as derived from multiple ordinal linear regression, is presented in Table 5.

While income was positively associated with* Pull* factor 1, it had a negative association with* Pull *factor 4. A better perceived health was positively associated with two of* Pull* factors, 2 and 4, and negatively associated with* Pull* factor 6. Employment was associated with* Pull *factor 2. While being a female was associated with* Pull *factor 6, a higher education was negatively associated with this factor. Factors 2 and 5 were not associated with any of the sociodemographic characteristics considered in this study.

## 4. Discussion

This study is the first attempt to understand the drivers of CAM use in the MENA region. It investigated the utilization of CAM using the Social Behavioral Model, in addition to the* Push* and* Pull* factors, in a national sample of Lebanese adults. The findings proposed an expanded version of the SBM, whereby the* Push* and* Pull* factors were integrated within the predisposing factors construct, as they have been proven to markedly influence health beliefs. Study findings further affirmed the utility of the SBM in explaining the use of CAM.

Analysis revealed that, compared to nonusers, significantly more CAM users belonged to the middle-aged group and to the highest household income category and reported having chronic diseases. In contrast, a significantly higher proportion of non-CAM users belonged to the higher education group. The finding with regard to age is in line with several reports revealing higher prevalence of CAM use among middle-aged adults [18, 24, 25]. Middle-aged adults are the bulk of the active workforce and are more likely to have a higher household income as compared to younger or older age groups, hence enabling access to CAM [2].

Among the health beliefs, the* Push* factor, dissatisfaction with conventional medicine, and three out of the six* Pull* factors studied were associated with CAM use. A systematic review of 87 studies examining CAM use in the European Union showed that the most common reason for CAM use was dissatisfaction with conventional care [26], which can arise from the belief that the latter may do more harm than good, may target one specific pathology ignoring the holistic good for the body, or may not be effective in curing certain diseases [27]. The advantage of regular assessment of patient satisfaction with treatment and services offered by healthcare providers and institutions does not only help improve the quality of services provided but also would prompt providers on the potential higher propensity of CAM use among dissatisfied patients [28]. On that front, it is important that healthcare providers systematically discuss CAM use with their patients, especially those with chronic diseases and those who express dissatisfaction with conventional treatment [27, 29].

In this study, the* Pull* factors associated with CAM use included the patients' preference for shared decision-making and assuming responsibility for own health rather than relying on the doctor (except for serious illnesses). The significant effect of these* Pull* factors in predicting CAM use was also highlighted in a systematic review, whereby the authors concluded that beliefs of the importance in participating in the treatment of oneself are important predictors of CAM use [9]. Such evidence suggested that CAM users want to actively participate in the treatment decisions and take some control over their own health. Enhancing patients' participation in decisions related to their health and empowering them to assume responsibility of their own health and wellbeing are mandatory for all systems endorsing a patient-centered approach of care [20].

Income as an enabling resource was also associated with CAM use. Corroborating these findings, several reports revealed that individuals with a higher income are more likely to be CAM users [30–32]. It is recommended that future studies examine in more detail the utilization patterns of CAM products across the various socioeconomic groups to better guide policy and practice recommendations. Such examinations can also advise on the enablers and barriers to CAM differentiated by average household income.

Furthermore, medical need (presence of chronic disease) was also associated with 46% increase in the odds of CAM use. Consistent with previous studies, indicators of poor health and chronic conditions have been reported to be associated with an increase in CAM use [33]. The standard operating procedures and clinical practice guidelines of healthcare providers need to integrate the probing of patients with chronic diseases on their use of CAM products. It is pivotal that the providers' approach acknowledges the value of CAM products if used properly rather than dismissing them in favor of conventional treatments [6]. This will minimize patients' resistance to disclosing CAM use and will help integrate CAM products and services into conventional treatment [34].

In this study, the* Push* factor (dissatisfaction with conventional medicine) was positively associated with younger age and with higher education and income levels. On the other hand, being a female, presence of chronic disease, and poor-to-good health status were associated with lower odds of dissatisfaction with conventional medicine. Younger individuals and those with higher education and income levels may have relatively higher expectations for quality and standards of care that make them more likely to be dissatisfied with conventional medicine [27]. They are also less frequent users of the healthcare system and thus will formulate their perspective based on sporadic episodes of care which give them a lower chance of building a trust relationship with their providers [35, 36]. In contrast, females, chronically ill patients, and those with poor-to-good health are more likely to be users of conventional healthcare services and will generally build better relationships with their providers. The frequent use and their reliance on conventional care may also help balance their expectations [37].

The association between sociodemographic and* Pull* factors also raises a number of interesting observations worthy of further investigations. For example, being a female was positively associated with the importance of faith and spirituality. Compared to males, females were more likely to rely on faith and spirituality [38]. Employed individuals were more likely to challenge the authority of their providers (*Pull* factor 2). This may be because employed individuals are relatively younger and are more likely to be healthy [37]. Lastly, income was positively associated with* Pull* factor 1 and negatively associated with preference to take care of own health rather than go to a doctor (except for serious illnesses). High-income individuals are more likely to have health insurance and coverage facilitating accessibility to health professionals and services whenever the need arises [24]. However, associated with such access is an expectation by high-income individuals for higher leverage in decisions related to health.

There are a number of shortcomings in this study which are worth noting. Investigating the drivers of CAM use by its various types, rather than a general category, may hold quite different sets of drivers of CAM use according to different types of CAM. In addition, in this study, the effects of the various constructs of the SBM and CAM use were studied using linear relationships. It is arguable that human behavior does not follow a linear relationship and is rather better explained using Chaos theory [39]. Furthermore, although this study focused on a prevalent CAM modality (biological-based therapies), future research is warranted in other branches of CAM, including “mind and body” and “energetic medicine.” Finally, our results indicated that women were more likely to use CAM (OR: 1.18, 95%CI: 0.94-1.47); however, these results were not significant. Future researches to further investigate the perception and attitude of women with regard to CAM use are warranted.

## 5. Conclusion

Given the propensity of CAM use, it is critical to reach a more comprehensive understanding of why people resort to it. The findings of this study affirmed the utility of the SBM in explaining the use of CAM among Lebanese adults and proposed a modified version of this model, whereby the* Push *and* Pull* factors are integrated as part of the predisposing factors. In summary, the results of this study indicated that, among the predisposing factors, age, the* Push* factor, and a number of the* Pull* factors were associated with CAM use. Specifically, older adults, those dissatisfied with conventional medicine (pushed), and those in favor of taking hold of one's health (pulled) were more likely to use CAM. Income emerged as an enabling factor, whereby a higher income was associated with more prevalent CAM use. The “presence of chronic illness” was the medical need variable significantly associated with CAM use. These findings are of importance to healthcare professionals, especially physicians, as they might help them understand the drivers of CAM use among their patients and improve patient-physician communication. Most importantly, formulating an evidence-based understanding of CAM use at the population level can help integrate CAM products and services into conventional treatment.

## Figures and Tables

**Table 1:** Sociodemographic characteristics of study participants (n = 1500).

	**Total**	**CAM Users**	**Non-CAM Users**	**Significance **
	N=1500	N=448	N=1052	
Age (years)				
32-51	588 (39.2)	198 (44.2)	390 (37.1)	*χ* ^2^ = 7.07, *p* = 0.03
≤31	543 (36.2)	153 (34.2)	390 (37.1)	
≥52	369 (24.6)	97 (21.7)	272 (25.9)	

Gender				
Males	816 (54.4)	231 (51.6)	585 (55.6)	*χ* ^2^ = 2.073, *p* = 0.15
Females	684 (45.6)	217 (48.4)	467 (44.4)	

Education				
Primary education or lower	337 (22.5)	112 (25.0)	225 (21.4)	*χ* ^2^ = 7.96, *p* = 0.02
High school/technical school	827 (55.1)	255 (56.9)	572 (54.4)	
University education or higher	336 (22.4)	81 (18.1)	255 (24.2)	

Employment status				
Unemployed	464 (30.9)	139 (31.0)	325 (30.9)	*χ* ^2^ = 0.003, *p* = 0.96
Employed	1036 (69.1)	309 (69.0)	727 (69.1)	

Monthly income ($)				
<1000	841 (56.1)	239 (53.3)	601 (57.2)	*χ* ^2^ = 7.08, *p* = 0.03
1000-2000	428 (28.5)	123 (27.5)	305 (29.0)	
>2000	231 (15.4)	86 (19.2)	145 (13.8)	

Presence of chronic disease				
No	1171 (78.1)	328 (73.2)	843 (80.1)	*χ* ^2^ = 8.784, *p* = 0.003
Yes	329 (21.9)	120 (26.8)	209 (19.9)	

Perceived health status				
Excellent	484 (32.3)	132 (29.5)	352 (33.5)	*χ* ^2^ = 5.360, *p* = 0.25
Very good	366 (24.4)	109 (24.3)	257 (24.4)	
Good	398 (26.5)	126 (28.1)	272 (25.9)	
Fair	200 (13.3)	69 (15.4)	131 (12.5)	
Poor	52 (3.5)	12 (2.7)	40 (3.8)	

**Table 2:** Health beliefs (including the *Push* and *Pull* factors) by CAM use among study participants (n = 1500).

**Push and pull factors**			**Total**	**CAM Users**	**Non-CAM Users**	Significance
			N=1500 (100%)	N=448 (29.9%)	N=1052 (70.1%)	
Push factors	During the past 12 months, have you felt dissatisfied with conventional medicine?	No	1217 (81.1)	319 (71.2)	898 (85.4)	*χ* ^2^ = 41.13, **p** < 0.0001
		Yes	283 (18.9)	129 (28.8)	154 (14.6)	

Pull factors	I prefer doctors give me choices or options & let me decide myself what to do	Strongly disagree	635 (42.3)	174 (38.8)	461 (43.8)	*χ* ^2^ = 51.17, **p** < 0.0001
		Disagree	121 (8.1)	61 (13.6)	60 (5.7)	
		Neither agree nor disagree	35 (2.3)	12 (2.7)	23 (2.2)	
		Agree	256 (17.1)	44 (9.8)	212 (20.2)	
		Strongly agree	453 (30.2)	157 (35.0)	296 (28.1)	
	Patients should challenge the authority of the doctor	Strongly disagree	1170 (78.0)	318 (71.0)	852 (81.0)	*χ* ^2^ = 30.67, , **p** < 0.0001
		Disagree	146 (9.7)	71 (15.8)	75 (7.1)	
		Neither agree nor disagree	37 (2.5)	14 (3.1)	23 (2.2)	
		Agree	87 (5.8)	24 (5.4)	63 (6.0)	
		Strongly agree	60 (4.0)	21 (4.7)	39 (3.7)	
	I prefer to assume some of the responsibility	Strongly disagree	1016 (67.7)	279 (62.3)	737 (70.1)	*χ* ^2^ = 16.46, **p** = 0.002
		Disagree	205 (13.7)	84 (18.8)	121 (11.5)	
		Neither agree nor disagree	76 (5.1)	19 (4.2)	57 (5.4)	
		Agree	112 (7.5)	37 (8.3)	75 (7.1)	
		Strongly agree	91 (6.1)	29 (6.5)	62 (5.9)	
	Except for serious illness, it is better to take care of your own health than go to the doctor	Strongly disagree	584 (38.9)	143 (31.9)	441 (41.9)	*χ* ^2^ = 56.94, , **p** < 0.0001
		Disagree	272 (18.1)	69 (15.4)	203 (19.3)	
		Neither agree nor disagree	47 (3.1)	22 (4.9)	25 (2.4)	
		Agree	259 (17.3)	123 (27.5)	136 (12.9)	
		Strongly agree	338 (22.5)	91 (20.3)	247 (23.5)	
	It is not obligatory to go to the doctor to treat oneself	Strongly disagree	1024 (68.3)	247 (55.1)	777 (73.9)	*χ* ^2^ = 60.87, , **p** < 0.0001
		Disagree	266 (17.7)	123 (27.5)	143 (13.6)	
		Neither agree nor disagree	38 (2.5)	15 (3.3)	23 (2.2)	
		Agree	96 (6.4)	42 (9.4)	54 (5.1)	
		Strongly agree	76 (5.1)	21 (4.7)	55 (5.2)	
	Spirituality and faith play important roles in life	Not important at all	62 (4.1)	17 (3.8)	45 (4.3)	*χ* ^2^ = 4.88, *p* = 0.300
		Not very important	47 (3.1)	11 (2.5)	36 (3.4)	
		Moderately important	188 (12.5)	64 (14.3)	124 (11.8)	
		Very important	1180 (78.7)	346 (77.2)	834 (79.3)	
		No answer	23 (1.5)	10 (2.2)	13 (1.2 )	

**Table 3:** Simple and multiple logistic regression analyses for the association of the various SBM constructs with the use of CAM.

**SBM constructs**			**OR (95**%** CI)**	**Adjusted OR (95**%** CI)**
**Predisposing characteristics**				

Sociodemographics	Age (years)	32-51	1.00	1.00
		≤31	**0.77 (0.60-0.99), p=0.05**	0.91 (0.69, 1.20), p=0.51
		≥52	**0.70 (0.53-0.94), p=0.02**	**0.61 (0.44, 0.86), p=0.005**
	Gender	Males	1.00	
		Females	1.18 (0.94-1.47), p=0.15	
	Education	Primary education or lower	1.00	1.00
		High school/technical school	0.90 (0.68-1.17), p=0.30	1.413 (0.72, 2.77), p=0.31
		Higher education	**0.64 (0.45-0.90)**, p**=0.04**	0.95 (0.46, 1.97), p=0.89
	Employment	Unemployed	1.00	
		Employed	0.99 (0.78-1.26), p=0.96	

Push factors	During the past 12 months, have you felt dissatisfied with conventional medicine?	No	1.00	1.00
		Yes	**2.36 (1.81-3.08), p<0.0001**	**2.45 (1.81, 3.32), p<0.0001**

Pull factors	I prefer doctors give me choices or options & let me decide myself what to do	Strongly disagree	1.00	1.00
		Disagree	**2.69 (1.81-4.00), p<0.0001**	**1.90 (1.21, 2.98), p=0.005**
		Neither agree nor disagree	1.38 (0.67-2.84), p=0.39	1.40 (0.63, 3.09), p=0.41
		Agree	**0.55 (0.38-0.79), p=0.001**	0.63 (0.42, 0.96), p=0.03
		Strongly agree	**1.40 (1.08-1.82), p=0.01**	**1.58 (1.19, 2.11), p=0.002**
	Patients should challenge the authority of the doctor	Strongly disagree	1.00	1.00
		Disagree	**2.54 (1.79-3.60)**,** p<0.0001**	1.56 (1.03, 2.37),p=0.04
		Neither agree nor disagree	1.63 (0.83-3.21), p=0.16	1.30 (0.60, 2.84), p=0.50
		Agree	1.02 (0.63-1.66), p=0.93	1.17 (0.67, 2.04), p=0.59
		Strongly agree	1.44 (0.84-2.49), p=0.19	1.39 (0.76, 2.52), p=0.28
	I prefer to assume some of the responsibility	Strongly disagree	1.00	1.00
		Disagree	**1.83 (1.34-2.50), p<0.0001**	1.06 (0.72, 1.55), p=0.78
		Neither agree nor disagree	0.88 (0.51-1.51), p=0.64	0.66 (0.36, 1.21), p=0.18
		Agree	1.30 (0.86-1.98), p=0.21	1.27 (0.80, 2.02), p=0.31
		Strongly agree	1.24 (0.78-1.96), p=0.37	1.16 (0.69, 1.95), p=0.57
	Except for serious illness, it is better to take care of your own health than go to the doctor	Strongly disagree	1.00	1.00
		Disagree	1.05 (0.752-1.461), p=0.781	1.37 (0.94, 1.99), p=0.10
		Neither agree nor disagree	**2.71 (1.48-4.96), p=0.01**	2.26 (1.14, 4.50),p=0.02
		Agree	**2.79 (2.05-3.80), p<0.0001**	**1.88(1.27, 2.78), p=0.002**
		Strongly agree	1.14 (0.84-1.54), p=0.41	1.30 (0.91, 1.85), p=0.14
	It is not obligatory to go to the doctor to treat oneself	Strongly disagree	1.00	1.00
		Disagree	**2.71 (2.04-3.58), p<0.0001**	**1.74 (1.20, 2.52), p=0.003**
		Neither agree nor disagree	**2.05 (1.054-3.993), p=0.034**	1.46 (0.68, 3.16), p=0.33
		Agree	**2.45 (1.595-3.753), p<0.0001**	1.74 (1.07, 2.81), p=0.02
		Strongly agree	1.20 (0.712-2.026), p=0.492	0.92 (0.51, 1.63), p=0.77
	Spirituality and faith play important roles in life	Not important at all	1.00	
		Not very important	0.81 (0.337-1.942), p=0.635	
		Moderately important	1.37 (0.725-2.576), p=0.335	
		Very important	1.10 (0.620-1.946), p=0.748	
		No answer	2.04 (0.752-5.510), p=0.162	

Enabling resources	Monthly income ($)	<$1000	1.00	1.00
		1000-2000	**1.50 (1.100-2.029)**, p**=0.04**	1.00 (0.75, 1.34), p=1.00
		>$2000	**1.47 (1.048-2.064), p=0.01**	**1.64 (1.16, 2.33), p=0.005**

Need factors	Presence of chronic disease	No	1.00	1.00
		Yes	**1.48 (1.140-1.910), p=0.003**	**1.46 (1.06, 2.02), p=0.01**
	Perceived health status	Excellent/Poor	1.00	
		Very good	1.15 (0.860-1.550), p=0.339	
		Good	1.26 (0.949-1.676), p=0.110	
		Fair	1.43 (1.012-2.032), p=0.043	

**Table 4:** Association of sociodemographic characteristics with the *Push* factor (dissatisfaction with conventional medicine) among study participants (n = 1500).

		**Crude OR**	**Adjusted OR**
		**(95 **%**CI)**	**(95 **%**CI)**
Age	32-51	Reference	Reference
	≤31	**2.49 (1.80, 3.46), p<0.0001**	**1.70 (1.19, 2.41), p=0.003**
	≥52	1.12 (0.82, 1.53), p=0.469	**1.97 (1.37,2.85), p=0.0003**

Gender	Males	Reference	Reference
	Females	**0.66 (0.51, 0.85), p=0.002**	0.821 (0.62, 1.09), p=0.17

Education	Primary education or lower	Reference	Reference
	High school/ technical school	**2.04 (1.17, 3.57), p=0.012**	1.20 (0.64, 2.24), p=0.57
	University education or higher	**3.69 (1.96, 6.93), p<0.0001**	1.44 (0.70,2.96), p=0.32

Employment	Unemployed	Reference	
	Employed	1.21 (0.92, 1.59), p=0.177	-

Monthly income ($)	<$1000	Reference	Reference
	1000-2000	**2.08 (1.51, 2.86), p<0.0001**	**1.80 (1.28, 2.52), p=0.001**
	>$2000	**3.86 (2.33, 6.41), p<0.0001**	**2.97 (1.73,5.08), p<0.0001 **

Presence of chronic disease	No	Reference	Reference
	Yes	**0.29 (0.22,0.38), p<0.001**	**0.49 (0.34, 0.71), p=0.0001 **

Perceived health status	Excellent	Reference	Reference
	Very good	0.86 (0.55, 1.36), p=0.528	0.97 (0.61, 1.55), p=0.89
	Good	**0.29 (0.19, 0.42), p<0.0001**	**0.37 (0.25, 0.56), p<0.0001**
	Fair	**0.18 (0.12, 0.27), p<0.0001**	**0.30 (0.19, 0.49), p<0.0001**
	Poor	**0.10 (0.05, 0.19), p<0.0001**	**0.21 (0.10, 0.42), p<0.0001**

**Table 5:** Significant associations among sociodemographic characteristics and the various *Pull* factors, as derived from ordinal multiple logistic regression^*∗*^.

Pull factors	Sociodemographic factors^*∗*^
(1) I prefer doctors give me choices or options & let me decide myself what to do	Income (+)
(2) Patients should challenge the authority of the doctor	Employment (+), perceived health (+)
(3) I prefer to assume some of the responsibility	-
(4) Except for serious illness, it is better to take care of your own health than go to the doctor	Income (-), perceived health (+)
(5) It is not obligatory to go to the doctor to treat oneself	-
(6) Spirituality and faith play important roles in life	Female (+), education (-), perceived health (-)

^*∗*^The associations summarized in this table are derived from Appendix A, which displays the parameter estimates table, which includes the coefficients, the 95% confidence interval of the coefficients, and their associated p-values for each of the *Pull* factors.

^*∗∗*^"-" indicates a negative association; "+" indicates a positive association.

## Data Availability

The data used to support the findings of this study are available from the corresponding author upon request.

## Supplementary Materials

Supplementary Materials Appendix A represents the associations of sociodemographic characteristics and each of the six* Pull* factors considered in this study, as derived from ordinal multiple logistic regression.
