# Supplementary material for: Understanding CAM Use in Lebanon: Findings from a National Survey
Source: Evid Based Complement Alternat Med. 2018 Jul 25;2018:4169159. doi: 10.1155/2018/4169159 (PMC6083547; doi:10.1155/2018/4169159)
Supplement: Supplementary Materials — Appendix A represents the associations of sociodemographic characteristics and each of the six Pull factors considered in this study, as derived from ordinal multiple logistic regression. [file 4169159.f1.docx]

**Appendix A**

**Table 6**. Associations of socio-demographic characteristics and the various Pull factors, as derived from ordinal multiple logistic regression.

|  | | **Pull factor 1**  Unadjusted estimate | **Unadjusted 95% CI** | **Significance**  **p-value** |  | **Pull factor 1**  Adjusted estimate | **Adjusted 95% CI** | **Significance**  **p-value** | |
| --- | --- | --- | --- | --- | --- | --- | --- | --- | --- |
| **Age** | 32-51 | Reference |  |  |  | Reference |  |  | |
|  | ≤31 | **0.22** | **[0.005,0.434]** | **0.045** |  | 0.152 | [-0.071,0.374] | | 0.182 |
|  | ≥52 | **0.31** | **[0.069,0.547]** | **0.012** |  | 0.295 | [0.049,0.542] | | 0.019 |
| **Gender** | Males | Reference |  |  |  | Reference |  | |  |
|  | Females | **-0.325** | **[-0.51,-0.14]** | **0.001** |  | -0.242 | [-0.435,-0.05] | | 0.014 |
| **Education** | Primary education or lower | Reference |  |  |  |  |  | |  |
|  | High school or technical school | -0.133 | [-0.61,0.35] | 0.588 |  |  | - | |  |
|  | Higher education | -0.049 | [-0.55,0.457] | 0.849 |  |  |  | |  |
| **Employment** | Unemployed | Reference |  |  |  |  |  | |  |
|  | Employed | 0.105 | [-0.09,0.306] | 0.308 |  |  | - | |  |
| **Income** | ˂$1000 | Reference |  |  |  | Reference |  | |  |
|  | 1000-2000 | -0.160 | [-0.37,0.55] | 0.145 |  | -0.154 | [-0.371,0.064] | | 0.166 |
|  | ˃$2000 | **0.623** | **[0.354,0.891]** | **p<0.0001** |  | **0.578** | **[0.304,0.853]** | | **<0.0001** |
| **Presence of chronic disease** | No | Reference |  |  |  |  |  | |  |
|  | Yes | 0.022 | [-0.20,0.25] | 0.849 |  |  | - | |  |
| **Perceived health status** | Excellent | Reference |  |  |  | Reference |  | |  |
|  | Very good | **-0.311** | **[-0.207,-0.880]** | **0.017** |  | -0.311 | [-0.567,-0.056] | | 0.017 |
|  | Good | -0.113 | [-0.548,0.096] | 0.382 |  | -0.113 | [-0.365,0.140] | | 0.382 |
|  | Fair | -0.226 | [-0.365, 0.140] | 0.169 |  | -0.226 | [-0.548,0.096] | | 0.169 |
|  | Poor | 0.336 | [-0.567,-0.056] | 0.225 |  | 0.336 | [-0.207,0.882] | | 0.225 |

|  | | **Pull factor 2**  Unadjusted estimate | **Unadjusted 95% CI** | **Significance**  **p-value** |  | **Pull factor 2**  Adjusted estimate | **Adjusted 95% CI** | **Significance**  **p-value** |
| --- | --- | --- | --- | --- | --- | --- | --- | --- |
| **Age** | 32-51 | Reference |  |  |  |  |  |  |
|  | ≤31 | -0.094 | [-0.374,0.187] | 0.885 |  |  | - |  |
|  | ≥52 | 0.023 | [-0.285,0.330] | 0.514 |  |  |  |  |
| **Gender** | Males | Reference |  |  |  |  |  |  |
|  | Females | -0.016 | [-0.259,0.227] | 0.898 |  |  | - |  |
| **Education** | Primary education or lower | Reference |  |  |  |  |  |  |
|  | High school or technical school | 0.539 | [-0.21,1.29] | 0.157 |  |  | - |  |
|  | Higher education | 0.663 | [-0.11,1.44] | 0.093 |  |  |  |  |
| **Employment** | Unemployed | Reference |  |  |  | Reference |  |  |
|  | Employed | **0.507** | **[0.225,0.789]** | **0.0004** |  | **0.547** | **[0.259,0.835]** | **0.0002** |
| **Income** | ˂$1000 | Reference |  |  |  |  |  |  |
|  | 1000-2000 | 0.109 | [-0.170,0.387] | 0.446 |  |  | - |  |
|  | ˃$2000 | 0.275 | [-0.060,0.611] | 0.108 |  |  |  |  |
| **Presence of chronic disease** | No | Reference |  |  |  |  |  |  |
|  | Yes | -0.001 | [-0.294,0.291] | 0.992 |  |  | - |  |
| **Perceived health status** | Excellent | Reference |  |  |  | Reference |  |  |
|  | Very good | **-0.311** | **[-0.207,-0.880]** | **0.017** |  | **0.579** | **[0.241,0.917]** | **0.001** |
|  | Good | -0.113 | [-0.548,0.096] | 0.382 |  | **0.532** | **[0.198,0.866]** | **0.002** |
|  | Fair | -0.226 | [-0.365, 0.140] | 0.169 |  | **0.832** | **[0.440,1.224]** | **<0.0001** |
|  | Poor | 0.336 | [-0.567,-0.056] | 0.225 |  | 0.248 | [-0.535,1.030] | 0.535 |

|  | | **Pull factor 3**  Unadjusted estimate | **Unadjusted 95% CI** | **Significance**  **p-value** |  | **Pull factor 3**  Adjusted estimate | **Adjusted 95% CI** | **Significance**  **p-value** |
| --- | --- | --- | --- | --- | --- | --- | --- | --- |
| **Age** | 32-51 | Reference |  |  |  |  |  |  |
|  | ≤31 | 0.134 | [-0.107,0.374] | 0.276 |  |  | - |  |
|  | ≥52 | -0.202 | [-0.483,0.078] | 0.158 |  |  |  |  |
| **Gender** | Males | Reference |  |  |  |  |  |  |
|  | Females | 0.033 | [-0.179,0.246] | 0.759 |  |  | - |  |
| **Education** | Primary education or lower | Reference |  |  |  |  |  |  |
|  | High school or technical school | -0.083 | [-0.634, 0.467] | 0.767 |  |  | - |  |
|  | Higher education | 0.329 | [-0.246, 0.905] | 0.262 |  |  |  |  |
| **Employment** | Unemployed | Reference |  |  |  |  |  |  |
|  | Employed | -0.019 | [-0.248,0.209] | 0.869 |  |  | - |  |
| **Income** | ˂$1000 | Reference |  |  |  |  |  |  |
|  | 1000-2000 | 0.164 | [-0.078,0.406] | 0.184 |  |  | - |  |
|  | ˃$2000 | 0.201 | [-0.099,0.501] | 0.189 |  |  |  |  |
| **Presence of chronic disease** | No | Reference |  |  |  |  |  |  |
|  | Yes | -0.193 | [-0.456,0.069] | 0.149 |  |  | - |  |
| **Perceived health status** | Excellent | Reference |  |  |  |  |  |  |
|  | Very good | 0.163 | [-0.120,-0.446] | 0.017 |  |  | - |  |
|  | Good | 0.072 | [-0.207,0.351] | 0.382 |  |  |  |  |
|  | Fair | 0.043 | [-0.305, 0.390] | 0.169 |  |  |  |  |
|  | Poor | 0.053 | [-0.548,-0.654] | 0.225 |  |  |  |  |

|  | | **Pull factor 4**  Unadjusted estimate | **Unadjusted**  **95% CI** | **Significance**  **p-value** |  | **Pull factor 4**  Adjusted estimate | **Adjusted**  **95% CI** | **Significance**  **p-value** |
| --- | --- | --- | --- | --- | --- | --- | --- | --- |
| **Age** | 32-51 | Reference |  |  |  | Reference |  |  |
|  | ≤31 | -0.172 | [-0.384,0.040] | 0.112 |  | -0.087 | [-0.307,0.133] | 0.437 |
|  | ≥52 | **0.255** | **[0.020,0.489]** | **0.033** |  | 0.203 | [-0.039,0.446] | 0.101 |
| **Gender** | Males | Reference |  |  |  |  |  |  |
|  | Females | -0.068 | [-0.252,0.116] | 0.469 |  |  | - |  |
| **Education** | Primary education or lower | Reference |  |  |  | Reference |  |  |
|  | High school or technical school | **-0.535** | **[-1.01,-0.064]** | **0.026** |  | -0.363 | [-0.941,114] | 0.145 |
|  | Higher education | **-0.735** | **[-1.234,-0.237]** | **0.004** |  | -0.414 | [-0.851,0.126] | 0.124 |
| **Employment** | Unemployed | Reference |  |  |  |  |  |  |
|  | Employed | 0.164 | [-0.035,0.362] | 0.106 |  |  | - |  |
| **Income** | ˂$1000 | Reference |  |  |  | Reference |  |  |
|  | 1000-2000 | **-0.307** | **[-0.518,-0.096]** | **0.004** |  | **-0.294** | **[-0.510,-0.078]** | **0.008** |
|  | ˃$2000 | **-0.554** | **[-0.823,-0.286]** | **p<0.0001** |  | **-0.478** | **[-0.756,-0.200]** | **0.001** |
| **Presence of chronic disease** | No | Reference |  |  |  |  |  |  |
|  | Yes | 0.200 | [-0.021,0.420] | 0.076 |  |  | - |  |
| **Perceived health status** | Excellent | Reference |  |  |  | Reference |  |  |
|  | Very good | **0.374** | **[0.127,0.621]** | **0.003** |  | **0.335** | **[0.084,0.586]** | **0.009** |
|  | Good | **0.368** | **[0.127,0.609]** | **0.003** |  | 0.284 | [0.035,0.532] | 0.025 |
|  | Fair | **0.477** | **[0.179, 0.775]** | **0.002** |  | 0.278 | [-0.037,0.593] | 0.083 |
|  | Poor | 0.284 | [-0.233,0.802] | 0.281 |  | -0.043 | [-0.584,0.499] | 0.877 |

|  | | **Pull factor 5**  Unadjusted estimate | **Unadjusted 95% CI** | **Significance**  **p-value** |  | **Pull factor 5**  Adjusted estimate | **Adjusted 95% CI** | **Significance**  **p-value** |
| --- | --- | --- | --- | --- | --- | --- | --- | --- |
| Age | 32-51 | Reference |  |  |  | Reference |  |  |
|  | ≤31 | 0.113 | [-0.128,0.354] | 0.359 |  | 0.120 | [-0.122,0.361] | 0.332 |
|  | ≥52 | **-0.321** | **[-0.606,-0.035]** | **0.028** |  | -0.280 | [-0.568,0.007] | 0.056 |
| Gender | Males | Reference |  |  |  |  |  |  |
|  | Females | -0.181 | [-0.396,0.034] | 0.099 |  |  | - |  |
| Education | Primary education or lower | Reference |  |  |  |  |  |  |
|  | High school or technical school | 0.013 | [-0.539,0.564] | 0.964 |  |  | - |  |
|  | Higher education | -0.045 | [-0.628,0.539] | 0.881 |  |  |  |  |
| Employment | Unemployed | Reference |  |  |  | Reference |  |  |
|  | Employed | **0.318** | **[0.080,0.556]** | **0.009** |  | 0.282 | [0.042,0.522] | 0.021 |
| Income | ˂$1000 | Reference |  |  |  |  |  |  |
|  | 1000-2000 | 0.193 | [-0.048,0.434] | 0.116 |  |  | - |  |
|  | ˃$2000 | -0.155 | [-0.474,0.163] | 0.339 |  |  |  |  |
| Presence of chronic disease | No | Reference |  |  |  |  |  |  |
|  | Yes | 0.060 | [-0.196,0.316] | 0.646 |  |  | - |  |
| Perceived health status | Excellent | Reference |  |  |  |  |  |  |
|  | Very good | 0.0.89 | [-0.198,0.375] | 0.544 |  |  | - |  |
|  | Good | 0.177 | [-0.100,0.454] | 0.211 |  |  |  |  |
|  | Fair | -0.092 | [-0.449, 0.264] | 0.613 |  |  |  |  |
|  | Poor | -0.225 | [-0.866,0.415] | 0.491 |  |  |  |  |

|  | | **Pull factor 6**  Unadjusted estimate | **Unadjusted 95% CI** | **Significance**  **p-value** |  | **Pull factor 6**  Adjusted estimate | **Adjusted**  **95% CI** | **Significance**  **p-value** |
| --- | --- | --- | --- | --- | --- | --- | --- | --- |
| Age | 32-51 | Reference |  |  |  | Reference |  |  |
|  | ≤31 | **-0.322** | **[-0.602,-0.042]** | **0.024** |  | -0.265 | [-0.559,0.029] | 0.077 |
|  | ≥52 | 0.056 | [-0.271,0.384] | 0.737 |  | 0.016 | [-0.327,0.360] | 0.926 |
| Gender | Males | Reference |  |  |  | Reference |  |  |
|  | Females | **0.641** | **[0.385,0.898]** | **p<0.0001** |  | **0.624** | **[0.342,0.906]** | **<0.0001** |
| Education | Primary education or lower | Reference |  |  |  | Reference |  |  |
|  | High school or technical school | **-0.883** | **[-1.675,-0.091** | **0.029** |  | -0.689 | [-1.494,0.116] | 0.093 |
|  | Higher education | **-1.264** | **[-2.081,-0.448]** | **0.002** |  | **-1.039** | **[-1.874,-0.203]** | **0.010** |
| Employment | Unemployed | Reference |  |  |  | Reference |  |  |
|  | Employed | **-0.401** | **[-0.680,-0.122]** | **0.005** |  | -0.067 | [-0.374,0.240] | 0.671 |
| Income | ˂$1000 | Reference |  |  |  |  |  |  |
|  | 1000-2000 | 0.175 | [-0.115,0.466] | 0.237 |  |  | - |  |
|  | ˃$2000 | -0.234 | [-0.571,0.102] | 0.172 |  |  |  |  |
| Presence of chronic disease | No | Reference |  |  |  |  |  |  |
|  | Yes | 0.079 | [-0.221,0.379] | 0.604 |  |  | - |  |
| Perceived health status | Excellent | Reference |  |  |  | Reference |  |  |
|  | Very good | **-0.438** | **[-0.758,-0.118]** | **0.007** |  | **-0.570** | **[-0.898,-0.242]** | **0.001** |
|  | Good | -0.150 | [-0.474,0.174] | 0.364 |  | -0.339 | [-0.676,-0.001] | 0.049 |
|  | Fair | -0.300 | [-0.136, 0.735] | 0.178 |  | 0.020 | [-0.437, 0.477] | 0.931 |
|  | Poor | 0.773 | [-0.075,1.621] | 0.074 |  | 0.267 | [-0.610,1.145] | 0.550 |
